# Development of a TaqMan-based triplex qPCR assay for the simultaneous detection of Muscovy reovirus, novel duck reovirus, and *Riemerella anatipestifer*

**DOI:** 10.3389/fvets.2026.1774735

**Published:** 2026-02-04

**Authors:** Zhihanlin Gan, Gaofeng Cai, Haiqin Li, Zhanhong Zheng, Linjie Song, Yiyang Zhang, Ping Liu, Yiwen Chen, Zhixuan Shen, Xiaona Gao, Xiaoquan Guo

**Affiliations:** 1 Jiangxi Provincial Key Laboratory for Animal Health, College of Animal Science and Technology, Jiangxi Agricultural University, Nanchang, Jiangxi, China; 2 Institute of Animal Husbandry and Veterinary Medicine, Jiangxi Academy of Agricultural Sciences, Nanchang, Jiangxi, China

**Keywords:** Muscovy duck reovirus, novel duck reovirus, *Riemerella anatifestifer*, TaqMan, triplex qPCR

## Abstract

**Introduction:**

In duck breeding production, the incidence of infection of Riemerella anatipestifer (RA), Muscovy duck reovirus (MDRV), and novel duck reovirus (NDRV) is gradually increasing. Because the clinical symptoms caused by these three pathogens are relatively similar, it is quite difficult to distinguish and diagnose.

**Methods:**

This study aims to establish a TaqMan-based triplex qPCR detection assay that can detect the above three pathogens at the same time. Specifically designed, optimized and verified specific primers and probes, targeting the dtxr gene of RA and the σB protein coding gene of MDRV and NDRV, respectively.

**Results:**

The results show that the assay has good specificity, sensitivity and stability. The linear detection range of the three pathogens is 102~109 copies/μL and the minimum detection limit can reach 101copies. In this study, 99 clinical on-site samples were tested, and positive samples were detected. In addition, through the analysis of the drug-resistant gene spectrum of the RA isolate, it was found that it carries aminoglycosides (RanA, RanB, aadS), macrocyclic esters [EreD, Erm(35), ErmF], chloramphenicol (floR) and tetracyclines [Tet(X3), Tet(X6), TetX] drug resistance genes of antibacterial drugs.

**Discussion:**

In summary, the TaqMan-based triplex qPCR detection assay established in this study can be used as a fast, sensitive and reliable technical means for large-scale monitoring of RA, MDRV and NDRV infection; at the same time, the analysis of the supporting drug-resistant gene detection assay can provide reference for the rational use of antibacterial drugs in duck breeding production. Test the basis, and then help the breeding link to take scientific and effective prevention and control intervention measures in a timely manner.

## 1 Introduction

*Riemerella anatifestifer* (RA) is a Gram-negative bacterium belonging to the Flavobacteriaceae family, which mainly infects waterfowl, causing acute sepsis and infectious inflammation of the heart and liver (1, 2). RA can be spread not only among waterfowl, but also to chickens. It is reported that Australia (3) and Greece (4) separated RA from the flock of chickens. There is also a case of RA transmission to chickens in China (5), mainly affecting the fallopian tubes of chickens, resulting in a decrease in egg production and a decrease incubation rate (6). At present, RA is spreading rapidly among Chinese chickens, posing a threat to the sustainable development of the poultry industry (7). In addition, RA often shows drug resistance, even multi-drug resistance (8) which makes it an important task to monitor its epidemiological characteristics and drug resistance levels.

Avian orthoreoviruses (ARVs) are classified as orthodontic viruses of the Reoviridae family, which pose a major health threat to waterfowl (9). Muscovy duck reovirus (MDRV) was first isolated in France in 1972 (10) and was later discovered in Israel, Germany and China (11, 12). MDRV mainly affects 2 to 4 weeks old musk ducks, resulting in hepatosplenomegaly and necrosis, with a high mortality rate (13). In 2005, a new variant called the novel duck reovirus (NDRV) was discovered in southeastern China. Compared with MDRV, it showed obvious epidemiological and pathogenic characteristics, such as severe liver necrosis and spleen hemorrhage (14, 15). Both MDRV and NDRV induce immunosuppression by damaging the central and peripheral immune organs, thus increasing the susceptibility of ducks to co-infection with bacterial pathogens (16).

In recent years, RA and waterfowl reovirus have been prevalent in China and caused economic losses (9, 14). Infections caused by RA, MDRV and NDRV show similar clinical manifestations, including hepatosplenic lesions and growth disorders, which makes it difficult to make differential diagnosis based solely on autopsy results (17). Existing diagnostic methods, such as quantitative PCR (qPCR) (18, 19), ELISA (20, 21), and CRISPR-Cas12a/Cas13a-based assays (22), are not enough to detect these three pathogens at the same time. The risk of co-infection and new virus strains in waterfowl populations is increasing.

In order to cope with this situation, we have developed a TaqMan-based triplex qPCR determination specifically for RA, MDRV and NDRV. By examining the conservative genome region of the reference strain and optimizing the primer-probe combination, the determination can quickly, sensitively and specifically identify all three pathogens in a single reaction. This assay provides a reliable diagnostic tool for the clinical diagnosis of the poultry industry.

## 2 Materials and methods

### 2.1 Viral strains and bacterial strains

NDRV, MDRV and RA strains used in this study were isolated from China and kept in our laboratory. Goose Astrovirus. Duck Hepatitis B Virus, *Escherichia coli*, and Salmonella isolated by our laboratory were used to assess specificity test.

### 2.2 Primers and probes

Considering the phylogenetic closeness between NDRV and MDRV, we developed species-specific primers and probes to differentiate these two pathogens without cross-reactivity. We retrieved all available genomic sequences of NDRV and MDRV from GenBank and performed sequence alignment using DNAMAN 9.0 (Lynnon Biosoft, USA). Through a comparative analysis of conserved regions, we identified the sigma B protein-encoding gene (σB) (NDRV: GenBank: PQ456210.1, MDRV: GenBank: KJ569581.1) as the target for detecting both viruses. For RA, the dtxr gene (GenBank: EU541215.1), a conserved regulator of iron metabolism, was selected as the amplification target. Three pairs of different primers and TaqMan probes were designed (Table 1). The TaqMan probe marked the FAM (MDRV), HEX (RA) and Cy5 (HEX) fluorescence reporting groups, respectively.

**Table 1:** Primers and probes applied in multiple detection.

Name	Sequences (5′ → 3′)	Length (bp)
NDRV-L	CGTGGATGGTCAAAGTCGTG	143
NDRV-R	CCAACGTGGGTAGTCTCCTC	
NDRV-probe	CY5-TCGCACTCCGCGGGCTCCAT-BHQ2	
MDRV-L	CGGGAAAGCCTGCTAAGTTC	97
MDRV-R	ACGTCATGGTCCCAATTGTC	
MDRV-probe	FAM-AGTCGGCATAACTGCTACCAACGGT-BHQ1	
RA-L	CCTCACGGAGAACCTATCCC	104
RA-R	ACAGCAGCTAGTGTTACCTT	
RA-probe	HEX-ACTCAGCGAATGTACCGAAGGCTCT-BHQ1	

### 2.3 Nucleic acid extraction

Use FastPure virus DNA/RNA Mini Kit v2 (Vazyme, Nanjing, China) to extract viral RNA. Use One Step PrimeScript™ RT-PCR kit (Takara, Beijing, China) for follow-up reverse transcription to synthesize complementary DNA (cDNA). Use HiPure bacterial DNA kit (Magen, Guangzhou, China) to isolate genomic DNA from bacterial cultures. All procedures are carried out strictly according to the instructions of the corresponding kit. RNA, cDNA and DNA are stored at −80 °C for subsequent analysis.

### 2.4 Standard plasmid construction

Using the cDNA of MDRV and NDRV and the DNA of RA as templates, use the corresponding primer to clone its gene fragments through PCR (Premix Taq; DL2, 000 DNA Marker, Takara, Beijing, China). Following recovery and purification, the amplified genes were individually ligated into the pClon007 vector to construct recombinant plasmids (TSINGKE, Beijing, China). The recombinant plasmids verified as correct through PCR and sequencing were designated as pRA, pNDRV, and pMDRV, respectively, and served as plasmid standards. The concentrations of these recombinant plasmids were measured using a Nanodrop spectrophotometer and subsequently converted to copy number (copies/μL).

### 2.5 Optimization of the singleplex RT-PCR

Single quantitative PCR (qPCR) assays were conducted utilizing the 2×Q3 Probe qPCR Master Mix (ToloBio, Shanghai, China) in accordance with the manufacturer’s instructions. An orthogonal matrix-based experimental design was employed to optimize the concentrations of primers and probes. Four final primer concentrations (0.1, 0.15, 0.2, and 0.25 pmol/μL) and seven probe concentrations (ranging from 0.1 to 0.4 pmol/μL in increments of 0.05 pmol/μL) were systematically evaluated. Identical plasmid standards (pRA, pNDRV, or pMDRV) served as templates for each experimental group. The thermal cycling protocol comprised an initial denaturation at 95 °C for 5 min, followed by 40 cycles of denaturation at 95 °C for 10 s and annealing/extension at 60 °C for 30 s.

### 2.6 Optimization of the TaqMan-based triplex qPCR

Utilizing the optimal primer-probe concentrations established from single RT-PCR (as detailed in Section 2.5), we integrated the three pairs of primer-probes into a unified triplex reaction system. The initial concentrations of primers and probes were proportionally adjusted to ensure consistency with the single conditions. The thermal cycling parameters adhered to the single protocol.

### 2.7 Standard curve

The three plasmid standards (pRA, pNDRV, pMDRV) underwent a 10-fold serial dilution in nuclease-free water to achieve concentrations ranging from 10^9^ to 10^2^ copies per microliter. Equal volumes of each diluted standard were then combined to formulate a triplex template mixture. Triplex PCR amplification was conducted in triplicate under the optimized reaction conditions detailed in Section 2.6. The cycle threshold (Ct) values were plotted against the logarithm of the template copy numbers (log₁₀ copies/μL) to construct standard curves.

### 2.8 Specificity test of the TaqMan-based triplex qPCR

The total RNA of MDRV, NDRV, and Goose Astrovirus was extracted and transcribed into cDNA. Total DNA from RA, Duck Hepatitis B Virus, *Escherichia coli*, and Salmonella was also extracted for specificity testing, ddH2O as negative controls.

### 2.9 Sensitivity test of the TaqMan-based triplex qPCR

A 1:1:1 mixture of plasmid standards (pMDRV, pNDRV, pRA; see Section 2.4) was subjected to serial 10-fold dilutions in nuclease-free water. Copy numbers were determined using the formula provided in Section 2.4, which is based on plasmid size and concentrations measured by NanoDrop. Triplex TaqMan qPCR amplifications were conducted under optimized conditions as detailed in Section 2.6. To compare sensitivity, the same dilution series was concurrently tested using conventional PCR with pathogen-specific primers (Table 1), and the amplification products were analyzed through 1.5% agarose gel electrophoresis.

### 2.10 Repeatability assessment of the TaqMan-based triplex qPCR

To assess the intra- and inter-assay reproducibility of the assay, we systematically conducted repeatability tests using standardized protocols. Three recombinant plasmid standards were uniformly mixed and subjected to serial 10-fold dilutions, resulting in final concentrations of 10^6^, 10^4^, and 10^2^ copies/μL. The repeatability of the assay was evaluated by calculating the standard deviation (SD) and coefficient of variation (CV) of three repeated measurements at different concentration levels.

### 2.11 Clinical samples collection

Cut the duck tissue sample into small pieces and put it into a 2 mL disinfection centrifuge tube. Add an appropriate amount of phosphate buffered brine (PBS) for grinding, and then freeze and thaw three times. Following centrifugation, total RNA and bacterial DNA were extracted from the supernatant. The total RNA was then reverse transcribed into complementary DNA (cDNA), which was analyzed using the established multiplex TaqMan fluorescence quantitative PCR assay.99 liver samples were collected from dead ducks (including Peking duck and Muscovy duck) in Jiangxi province in 2025.

### 2.12 Detection of drug resistance genes in RA isolates

Bacterial isolation was performed from RA-positive samples. Single colonies suspected to be RA were subjected to detection using the described assay. Strains testing positive for RA were further analyzed by PCR using RA-16sRNA primers, and the resulting products were sequenced for definitive identification. After confirmation as RA, the isolates were screened via PCR with primers (Table 2).

**Table 2:** Primers used in bacterial resistance genes detection.

Name	ARO_ID	Sequences (5′ → 3′)	Length (bp)
RanA-F	3005091	GCATTATGGGACGAACTG	552
RanA-R		TTGTTTCCCTTACTTGCT	
RanB-F	3005090	TACCATTCCCATTCCACT	288
RanB-R		CCTGCTAAATAACCTCCAT	
floR-F	3002705	AAATAGTAACCGAAGCCACCACA	437
floR-R		GCCGTAGATGACGACACCCT	
EreD-F	3004608	TTGCCCACAATGCTCACA	149
EreD-R		CAATGCCGCAGTTTCTCC	
aads-F	3004683	AAACCGTTATTCGCACAG	134
aads-R		TTTAGCCCACATTTCCTG	
Erm(35)-F	3000604	GGGTTCCTTACTGTCCAT	644
Erm(35)-R		ACAACTTCCAGCATTTCTA	
floR-F	3002705	TGCTGCTGATGGCTCCTTTC	104
floR-R		CATAGCGGGCGTCGTGTT	
ErmF-F	3000498	TGGCATTACTTCCGATAT	460
ErmF-R		GACAACTTCCAGCATTTC	
Tet(X6)-F	3005056	ACCGTCATTTGGGATAGA	587
Tet(X6)-R		CCACTGTTTACGCCTTGT	
TetX-F	3000205	GCGAATAGATACAGACAAACA	431
TetX-R		TCTTACCAGGTTCAAGCATA	
Tet(X3)-F	3004719	AAACAACCACGGGACAT	389
Tet(X3)-R		TTTCACTTCAAACGCATAG	
TetX-F	3000205	GTGGACCCGTTGGACT	408
TetX-R		TTTCACTCGGTTTATTCTC	
TetX-F	3000205	GTGGACCCGTTGGACT	758
TetX-R		TTCTTTGTAGCGTTCGTC	

## 3 Results

### 3.1 Development and optimization of the TaqMan-based triplex qPCR

The results of optimizing the ratio of primers and probes in a single detection reaction (Table 3) illustrates variations in reaction performance across different probe and primer ratios. Considering both reaction efficiency and cost-effectiveness, the optimized 20 μL qPCR system comprises 10 μL of 2×Q3 probe qPCR Master Mix, 0.4 μL of NDRV-L/R, 0.4 μL of NDRV-probe, 0.3 μL of MDRV-L/R, 0.4 μL of MDRV-probe, 0.4 μL of RA-L/R, and 0.4 μL of RA-probe, with the volume adjusted to 20 μL using nuclease-free water.

**Table 3:** Optimization of the TaqMan-based triplex qPCR assay.

Target	pmol/μL	Probe primer	0.10	0.15	0.20	0.25	0.30	0.35	0.40
NDRV	0.10		14.45	14.91	14.41	14.71	15.16	14.83	13.07
	0.15		13.05	13.64	13.15	13.10	13.72	13.69	11.65
	0.20		12.28	12.94	12.46	12.19	12.60	12.93	11.73
	0.25		12.15	12.32	12.49	12.66	12.57	12.54	11.76
MDRV	0.10		19.64	19.70	19.48	19.48	19.69	19.64	19.64
	0.15		18.27	18.24	18.27	18.25	18.03	18.22	19.50
	0.20		17.56	17.41	17.15	17.60	17.49	17.23	17.63
	0.25		18.00	17.84	17.80	17.28	17.75	17.89	17.73
RA	0.10		21.23	20.93	20.63	20.40	21.06	21.34	20.34
	0.15		19.98	19.66	19.57	19.66	19.42	19.32	19.31
	0.20		18.73	18.85	18.62	18.21	18.63	18.56	18.50
	0.25		19.22	18.90	18.71	18.87	18.68	18.60	18.15

### 3.2 Standard curves

**Figure 1:**
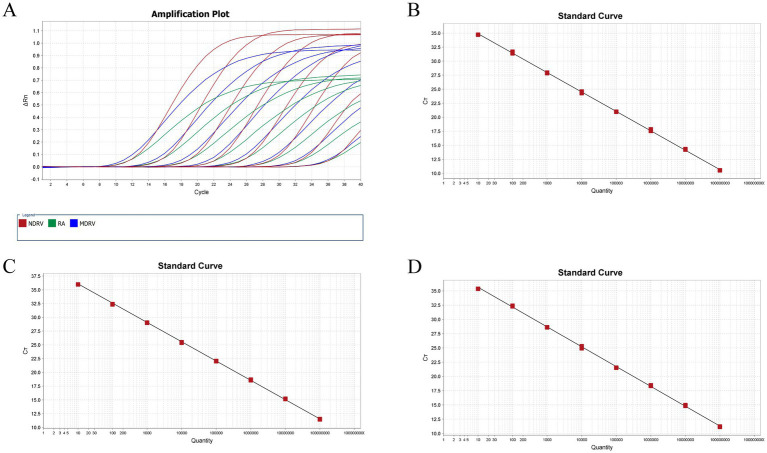
Establishment of standard curve. **(A) A**mplification curve of plasmid standard with concentration of 10^9^~10^2^ copies/μL and negative control. **(B)** MDRV standard curve: Slope: −3.463, Y-Inter: 38.354, Eff%: 94.424, *R*^2^: 0.999. **(C)** NDRV standard curve: Slope: −3.540, Y-Inter: 39.576, Eff%: 92.932, *R*^2^: 0.999. **(D)** RA standard curve: Slope: −3.477, Y-Inter: 39.105, Eff%: 93.912, *R*^2^: 0.999.

The recombinant plasmid standards, pRA, pNDRV, and pMDRV, were subjected to a 10-fold serial dilution and subsequently combined in equal volumes. Plasmids with final concentrations ranging from 10^9^ copies/μL to 10^2^ copies/μL were utilized as templates. The TaqMan-based triplex qPCR amplification curves and standard curves were generated using the methodology developed in this study. The correlation coefficients (*R*^2^) of the three standard curves exceeded 0.990. The findings indicated a strong linear relationship between the concentrations of 10^9^ copies/μL to 10^2^ copies/μL and the Ct values of the three plasmid standards (Figure 1).

### 3.3 Specificity

**Figure 2:**
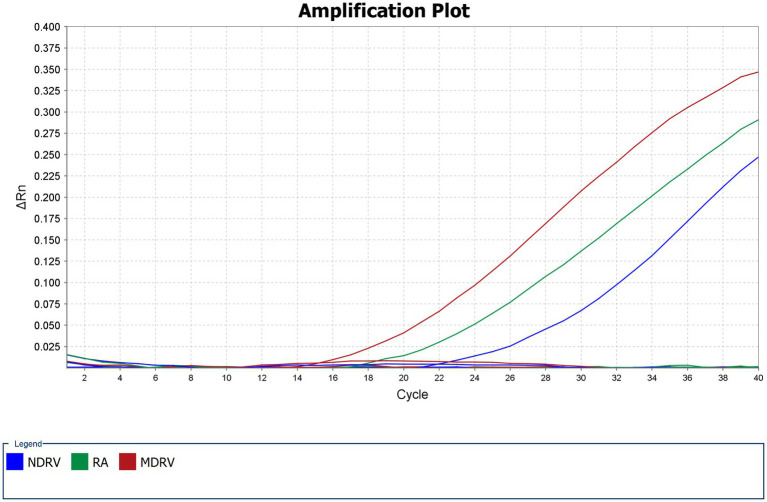
Specificity test. Only the target pathogen produced an amplification curve.

The results (Figure 2) showed that only RA, NDRV, and MDRV exhibited specific amplification curves, while other pathogens failed to produce amplification. This demonstrates the specificity of the primers and probes.

### 3.4 Sensitivity

**Figure 3:**
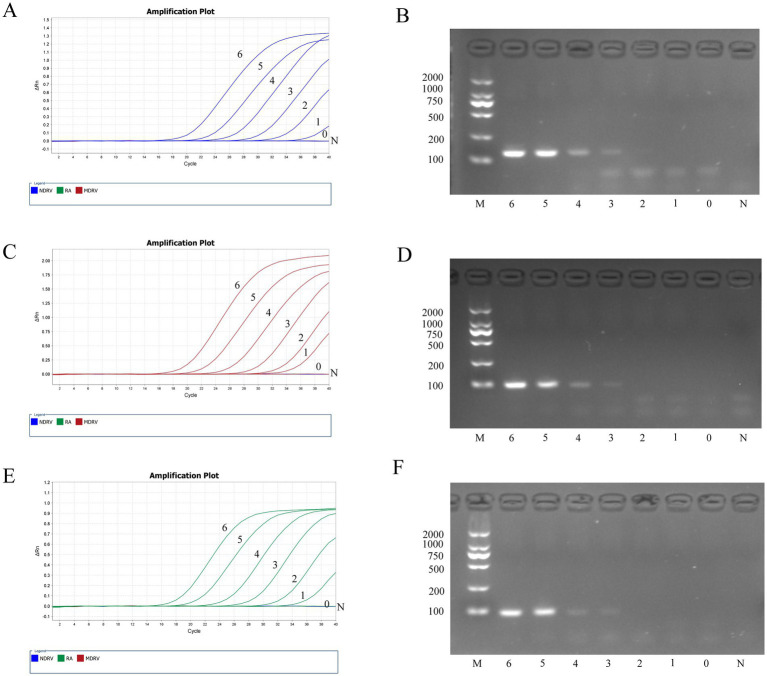
Sensitivity test of the method and common PCR method. **(A,B)**-NDRV standards, **(C,D)**-MDRV standards, **(E,F)**-RA standards. 6–0 are plasmid standards with the concentration of 10^6^–10^0^ copies/μL in turn, N is negative control, M is marker.

Sensitivity assessments of both the TaqMan-based triplex qPCR and PCR were performed utilizing serial 10-fold dilutions of plasmid standards (pRA, pNDRV, and pMDRV) combined in equal volume ratios. Quantitative analysis determined the limits of detection to be 10^1^ copies/μL for pRA, 10^1^ copies/μL for pNDRV, and 10^1^ copies/μL for pMDRV when employing our triplex assay. In contrast, the conventional PCR exhibited LODs of 10^3^ copies/μL, 10^3^ copies/μL, and 10^3^ copies/μL, respectively. The TaqMan-based triplex qPCR developed herein demonstrated a 100-fold increase in sensitivity across all targets, underscoring the superior specificity and sensitivity attributes characteristic of TaqMan probe chemistry (Figure 3).

### 3.5 Repeatability and reproducibility

The results show that in the intra-group and inter-group repeatability test, the variation coefficient of the Ct value is less than 2.5% (Table 4), indicating that the assay has good repeatability.

**Table 4:** Repeatability and reproducibility of the assay.

Plasmid standard	Conc (copies/μL)	Intra-group			Inter-group		
		Mean	SD	CV (%)	Mean	SD	CV (%)
pMDRV	10^6^	21.13	0.19	0.92	21.38	0.18	0.84
	10^4^	26.47	0.08	0.30	25.86	0.43	1.63
	10^2^	34.37	0.44	1.28	33.87	0.35	1.04
pNDRV	10^6^	19.44	0.18	0.93	19.55	0.08	0.39
	10^4^	24.99	0.04	0.16	24.27	0.51	2.06
	10^2^	33.14	0.37	1.12	32.50	0.45	1.38
pRA	10^6^	22.19	0.20	0.90	22.53	0.24	1.08
	10^4^	27.52	0.11	0.39	27.00	0.37	1.35
	10^2^	35.56	0.29	0.82	35.17	0.27	0.77

### 3.6 Application and detection of clinical samples

**Figure 4:**
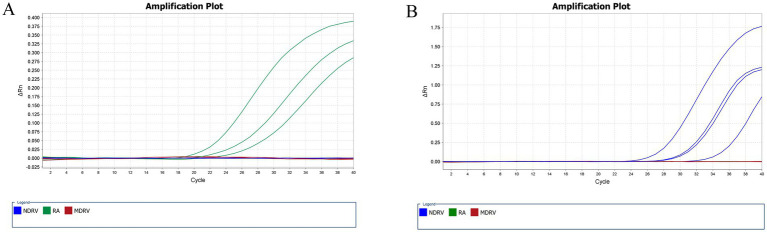
Positive sample in clinical detection. Amplification plot of NDRV **(A)** and RA **(B)** positive samples.

To further substantiate the clinical applicability of this assay, we conducted an analysis of 99 liver samples obtained from ducks with suspected infections across various regions. Pathogen-specific detection yielded positive results in 28 samples (Figure 4), RA (*n* = 8), NDRV (*n* = 20), MDRV (*n* = 0). These results confirm the prevalence of infections in field settings. The assay demonstrated significant diagnostic utility for epidemiological surveillance and clinical management of waterfowl diseases.

### 3.7 Detection of drug resistance genes in RA isolates

**Figure 5:**
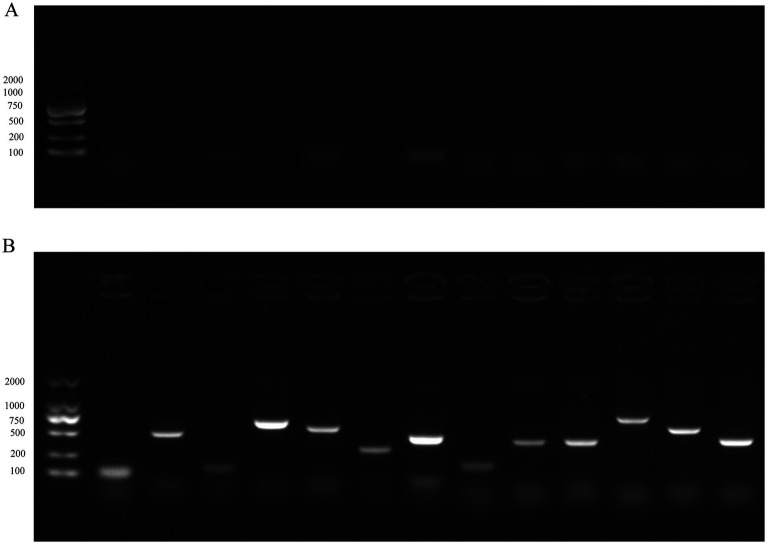
Detection of drug resistance genes of isolated strains. **(A)** Negative control **(B)** This strain carries multiple drug resistance genes.

The presence of amplified bands indicated potential carriage of the corresponding resistance genes. The detection results of resistance genes in one representative isolate, demonstrating that this strain carries multiple antibiotic resistance genes (Figure 5). These include genes conferring resistance to aminoglycoside antibiotics (RanA, RanB, and aads), macrolide resistance genes [EreD, Erm(35), and ErmF], a chloramphenicol resistance gene (floR), and tetracycline resistance genes [Tet(X3), Tet(X6), and TetX]. And strains carrying a variety of resistance genes accounted for a high proportion in this trial (Table 5).

**Table 5:** Results of bacterial resistance genes detection.

Name	Positive samples	Name	Positive samples
RanA	5/5	Erm(35)	5/5
RanB	5/5	ErmF	5/5
floR	5/5	Tet(X6)	5/5
EreD	4/5	Tet(X3)	5/5
aads	4/5	TetX	5/5

## 4 Discussion

The duck farming industry faces significant threats from concurrent infections involving RA, MDRV, and NDRV. These pathogens contribute to substantial economic losses globally. Infection is usually manifested as similar clinical symptoms and pathological changes, and it is difficult to distinguish and diagnose through autopsy alone. Therefore, an accurate molecular detection assay is urgently needed.

In this study, we established a TaqMan-based triplex qPCR to detect RA, MDRV and NDRV at the same time. We designed a primer probe for the highly conservative dtxr gene of RA and the σB protein coding genes of MDRV and NDRV. Both the probes and primers are designed on the conserved sequence of the pathogen and can match most known pathogen strains. By using three different fluorescent reports (CY5, FAM, HEX), this assay can efficiently distinguish three pathogens in a single reaction at the same time, thus reducing the risk of cross-contamination and improving detection efficiency. The assay has specificity, good stability (in-test CV ≤ 1.28%, inter-test CV ≤ 2.06%) and high sensitivity, and can detect 10^1^ copies. The sensitivity is comparable or better than that of other previously published PCR assays (23, 24), simpler than the ELISA method (20, 21) and directly detects pathogens, avoiding the interference of complex serum backgrounds.

The clinical samples collected in this experiment are all livers of sick ducks, with obvious liver lesions. We tested these samples, and some of them detected RA or NDRV. The remaining samples may be caused by other pathogen infections, which to a certain extent reflects the function of accurate identification and diagnosis of this method. The drug resistance phenotype of RA is highly correlated with the drug resistance genotype, it has been previously reported that drug resistance is widespread among the prevalent strains (25, 26), and the monitoring of drug resistance genes plays an important role in drug selection and drug resistance research. Previous studies have found a variety of resistance genes in RA, which make RA resistant to a variety of antibiotics such as aminoglycosides, erythromycin, etc. (27–30). Our resistance gene detection confirmed multidrug-resistant (MDR) RA strains carrying genes conferring resistance to aminoglycosides (RanA, RanB, aads), macrolides [EreD, Erm(35), ErmF], chloramphenicol (floR), and tetracyclines [Tet(X3), Tet(X6), TetX]. The emergence of these MDR strains is likely facilitated by mobile genetic elements (e.g., plasmids, transposons), which can horizontally transfer resistance genes. In this study, the results of drug-resistant gene detection of isolated strains show that the proportion of multiple drug-resistant genes carried by current epidemic strains is very high. When viral infections such as NDRV damage the host’s immunity, effective antibiotics are crucial to controlling secondary bacterial pathogens. Bacterial multidrug resistance may lead to treatment failure and significant economic losses. It is worth noting that no MDRV was detected in the 99 clinical samples analyzed, in contrast to the observed infection of RA and NDRV. This may be due to the fact that the MDRV host profile is not as extensive as NDRV, mainly susceptible to Muscovy ducks and half Muscovy ducks, there are mature commercial vaccines, many farms include them in immunization procedures, and standardized vaccine use largely limits the prevalence of MDRV. However, no detection is not the same as the disappearance of the virus. The virus may continue to evolve under immune pressure, eventually the emergence of mutant strains and epidemics, so continuous monitoring of MDRV is still required.

This method has many advantages, but also disadvantages. For example, it is necessary to extract viral RNA and bacterial DNA separately, which requires more work. The increase in steps not only prolongs the testing time, but also correspondingly increases the risk of cross-contamination between samples or environmental nucleic acid contamination. Therefore, this puts forward stricter requirements for the cleanliness of the laboratory environment, the operation specifications of experimenters, and the quality control of the process. Going forward, we will focus on developing an integrated assay solution that is designed to enable easier and faster operation, while also providing high-throughput detection capabilities for RNA viruses and bacteria. In a word, the method developed in this paper provides a fast, sensitive and reliable tool for simultaneous detection and identification of RA, MDRV and NDRV.

## Data Availability

The data that support the findings of this study are available from the corresponding authors.
